# Giant Aortic Arch Aneurysm Presenting as Ortner's Syndrome in Polycystic Kidney Disease: A Radiological Perspective

**DOI:** 10.7759/cureus.63490

**Published:** 2024-06-29

**Authors:** Arvinder Wander, Vandeep S Basra, Tejinder S Malhi, Ramandeep Singh

**Affiliations:** 1 Pediatrics, All India Institute of Medical Sciences, Bathinda, Bathinda, IND; 2 Cardiology, All India Institute of Medical Sciences, Bathinda, Bathinda, IND; 3 Radiodiagnosis, All India Institute of Medical Sciences, Bathinda, Bathinda, IND

**Keywords:** ortner's syndrome, pulmonary oligemia, ct aortogram, polycystic kidney disease, giant aortic arch aneurysm

## Abstract

Polycystic kidney disease (PKD) is a genetic disease characterized by the formation of multiple cysts in bilateral kidneys. While renal complications are predominant, cardiovascular manifestations such as aortic aneurysms can also occur. Although there are a few case reports of giant aortic arch aneurysms, to the best of our knowledge, this has been rarely reported in patients with PKD. Additionally, the clinical presentation of the index case is unique.

## Introduction

Autosomal dominant polycystic kidney disease (ADPKD) is an inherited disease characterized by the formation of multiple cysts in bilateral kidneys and less commonly in the liver, pancreas, and spleen [1]. As polycystin has been demonstrated to support the preservation of arterial integrity, various vascular abnormalities such as intracranial aneurysms, aortic aneurysms, and dissection of the coronary and vertebral arteries are associated with ADPKD [2].

Aortic arch aneurysms are uncommon and account for <10% of thoracic aortic aneurysms [3]. This article delineates an unusual scenario where a PKD-diagnosed individual manifested pressure symptoms due to a giant aortic arch aneurysm. To the best of our knowledge, giant aortic arch aneurysm is rarely reported in PKD patients.

## Case presentation

A 58-year-old male with a known case of PKD presented with a change in voice and dysphagia for three months. He also complained of progressively worsening dyspnea on exertion and occasional chest discomfort for the past two months. He was hypertensive, which was fairly controlled on two antihypertensive drugs, namely telmisartan and amlodipine. He was a non-smoker and non-diabetic. Family history revealed PKD in his younger brother with no known cardiovascular disorders. Peripheral pulses were normal. Blood pressure was 140/96 mmHg in both arms. Cardiac auscultation revealed normal S1 and S2 heart sounds with no murmurs. Blood investigations (Table 1) such as complete blood count and lipid profile were normal except for mildly raised serum creatinine levels (1.4 mg/dL). The patient was referred to an ear, nose, and throat (ENT) surgeon for a laryngoscopy, which revealed left vocal cord palsy; however, no laryngeal growth was noted.

**Table 1:** Blood investigations of the patient

Investigation	Result	Unit	Reference range
White blood cells	6.45	x10^3^ /μl	4.00-11.00
RBC	4.71	x10^6^ /μl	3.50-5.50
Hemoglobin	12.9	gm/dl	11.0-16.0
Platelet	190	x10^3^ /μl	150-450
Glycosylated hemoglobin	5.5	%	Normal < 5.7; prediabetic 5.7-6.4; diabetic > 6.4
Urea	60	mg/dl	15-45
Creatinine	1.4	mg/dl	0.6-1.10
Cholesterol	192	mg/dl	<200
Triglyceride	148	mg/dl	<150

Imaging

**Figure 1:**
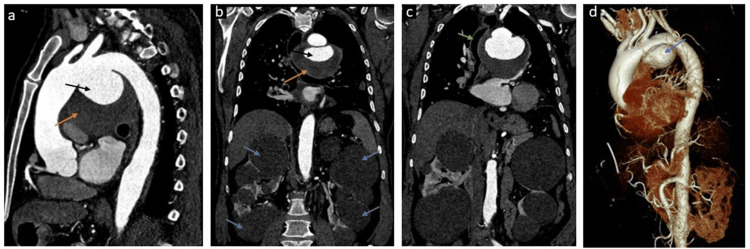
Computed tomography angiograms demonstrating a giant aortic arch aneurysm in polycystic kidney disease patient (A) Sagittal image demonstrating a large aneurysm from the inferior surface of the arch of aorta (black arrow) and large intramural thrombus (orange arrow). (B) Coronal image demonstrating the same (black and orange arrows) and multiple variable-sized bilateral simple renal cortical cysts (blue arrows). (C) Coronal image showing rightward tracheal shift (green arrow) due to mass effect by the aneurysm. (D) 3D virtual rendering technique (VRT) image demonstrating aortic arch aneurysm (blue arrow).

Chest X-ray showed a large mediastinal opacity in the upper and mid-zones, causing right-sided tracheal shift. There was also gross enlargement of the aortic knob. Contrast-enhanced computed tomography (CECT) chest with aortogram was ordered to confirm the diagnosis. A contrast-enhanced computed tomography (CT) angiogram demonstrated a large saccular aneurysm arising from the inferior surface of the aortic arch, extending from the origin of the left common carotid artery to the descending thoracic aorta. It was partially thrombosed with a maximum external diameter of 9.5 cm and a maximum internal diameter of 6.8 cm (Figure 1).

**Figure 2:**
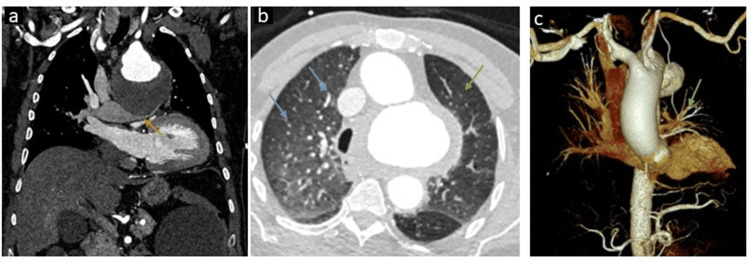
Computed tomography demonstrating the mass effect on the left main pulmonary artery with resultant pulmonary oligemia (A) Coronal image demonstrating severe compression of the left main pulmonary artery (yellow arrow) by the giant aortic arch aneurysm. (B) Axial image in lung window at the level of bilateral upper lobes showing a reduced caliber of the segmental and subsegmental pulmonary arteries, with resultant hyperlucent left lung parenchyma suggesting oligemia (green arrow). Note the normal-sized right upper lobe segmental and subsegmental pulmonary arteries (blue arrows). (C) 3D virtual rendering technique (VRT) image demonstrating a marked reduction in the size of the left pulmonary vasculature (green arrow) at the level of the hilum due to compression of the left main pulmonary artery.

The aneurysm was causing severe compression on the left pulmonary artery with resultant relative pulmonary oligemia on the left side (Figure 2).

There was significant compression on the mid-thoracic esophagus and left recurrent laryngeal nerve. The trachea was shifted toward the right side; however, no significant luminal narrowing was seen. There was no evidence of significant atherosclerotic changes in the aorta and its branches. A note was made of multiple large simple cysts in bilateral kidneys. No aneurysm of coronary arteries or abdominal aorta or its branch was seen. Time of flight MR angiography of the brain revealed no intracranial aneurysm. CT reporting template for aortic arch aneurysm is shown in Table 2.

**Table 2:** CT reporting template for aortic arch aneurysm

Morphological features
Total sac dimension (outer surface to outer surface)
Patent sac dimension (in case of mural thrombus)
Shape: fusiform or saccular
Mural characteristics
Presence of calcification
Presence of mural thrombus
Mural thickening
Atherosclerotic plaques
Location and relationship to aortic arch branches
Diameter of aorta proximal and distal to aneurysm
Location in terms of anatomical landing zones of aorta
Relation of the aneurysm with origins of aortic arch branches
Characterization of possible etiology
True versus pseudoaneurysm
Clue for mycotic aneurysm— adjacent soft tissue stranding, collection
Atherosclerotic changes in the aorta
Complications
Signs of impending rupture
Leak
Thrombosis

Outcome and follow-up

Given the size and location of the aneurysm, surgical intervention was deemed necessary. Hence, the patient was advised for surgical management of the aneurysm, but he refused at that time. However, he presented again after one month with acute chest pain, and, unfortunately, succumbed to acute aortic syndrome before any further management could be initiated.

## Discussion

PKD predisposes patients to various cardiovascular complications, including intracranial aneurysms, mitral valve prolapse, and aortic aneurysms. The exact pathogenesis is unclear, but it is believed to be multifactorial gameplay involving genetic and hemodynamic factors. The presence of aortic aneurysms is uncommon in ADPKD patients and mostly involves the abdominal aorta [2]. The presence of hoarseness of voice and dysphagia due to the mass effect of giant aortic arch aneurysms in PKD patients makes our case really unique because such a combination has been rarely reported to date. Hoarseness of voice is due to the left recurrent laryngeal nerve compression as it courses adjacent to the aortic arch. Hoarseness occurring in the setting of cardiovascular cause is termed cardiovocal or Ortner's syndrome. This syndrome is associated with many cardiovascular diseases such as mitral valve disease, severe pulmonary hypertension, or congenital diseases [4]. However, it is rarely caused by an aortic aneurysm [4]. Dysphagia in the index cause was the result of compression on the esophagus by the aortic arch aneurysm. Cardiovocal syndrome, together with the presence of dysphagia, dyspnea, and chest pain in patients with aortic arch aneurysm, is a surgical indication for the treatment of aortic aneurysms [5].

Diagnosis of the aortic arch aneurysm was relatively late in the index case posing various clinical and surgical issues and ultimately patient morbidity. This highlights the importance of screening in ADPKD patients for early detection of aneurysms. Though screening for intracranial aneurysms is becoming a common practice in ADPKD patients, screening for aortic and coronary aneurysms is not frequent because they are uncommon. Due to their rarity, screening may not be appropriate for all ADPKD patients, but drawing special attention and timely screening of patients with additional risk factors such as hypertension, age > 60 years, and chest discomfort, might prevent grave prognosis. The other point to note is that the patient was asymptomatic until the last three months despite the large size of the aneurysm; therefore, even asymptomatic elderly patients of PKD may benefit from the screening.

According to the guidelines by the National Institute for Health and Care Excellence, screening for aortic aneurysm is recommended for all men aged 66 years and above or if there is a presence of risk factors such as hypertension. Recommendations for surgical and endovascular management of aortic arch aneurysms are given in Table 3 [5].

**Table 3:** Recommendations for surgical and endovascular management of aortic arch aneurysms Source: Ref. [5].

Strength of recommendation	Recommendations
Strong (benefit >>> risk)	In patients with an aortic arch aneurysm who have symptoms attributable to the aneurysm and are at low or intermediate operative risk, open surgical replacement is recommended.
Moderate (benefit >> risk)	In patients with an isolated aortic arch aneurysm who are asymptomatic and have a low operative risk, open surgical replacement at an arch diameter of ≥5.5 cm is reasonable.
Weak (benefit >/= risk)	In patients with an aortic arch aneurysm who are asymptomatic but meet criteria for intervention, but have a high risk from open surgical repair, a hybrid or endovascular approach may be reasonable.

## Conclusions

This case underscores the importance of recognizing cardiovascular manifestations in patients with PKD. A high index of suspicion, coupled with prompt diagnostic evaluation and multidisciplinary management, can lead to favorable outcomes in complex cases like giant aortic arch aneurysms. Early detection and intervention are paramount due to the increased risk of rupture associated with giant aortic arch aneurysms. Surgical repair remains the mainstay of treatment, although endovascular techniques may be considered in selected cases.
